# Innovative Digital Phenotyping Method to Assess Body Representations in Autistic Adults: A Perspective on Multisensor Evaluation

**DOI:** 10.3390/s24206523

**Published:** 2024-10-10

**Authors:** Joanna Mourad, Kim Daniels, Katleen Bogaerts, Martin Desseilles, Bruno Bonnechère

**Affiliations:** 1 REVAL Rehabilitation Research Center, Faculty of Rehabilitation Sciences, Hasselt University, 3590 Diepenbeek, Belgium; joanna.mourad@uhasselt.be (J.M.); kim.daniels@pxl.be (K.D.); katleen.bogaerts@uhasselt.be (K.B.); 2 Technology-Supported and Data-Driven Rehabilitation, Data Sciences Institute, Hasselt University, 3590 Diepenbeek, Belgium; 3 Department of Psychology, University of Namur, 5000 Namur, Belgium; martin.desseilles@unamur.be; 4 Transition Institute, University of Namur, 5000 Namur, Belgium; 5 Department of PXL—Healthcare, PXL University of Applied Sciences and Arts, 3500 Hasselt, Belgium; 6 Health Psychology, Faculty of Psychology and Educational Sciences, University of Leuven, 3000 Leuven, Belgium

**Keywords:** body representations, assessment, autistic adults, multisensor, data integration, digital phenotyping

## Abstract

In this perspective paper, we propose a novel tech-driven method to evaluate body representations (BRs) in autistic individuals. Our goal is to deepen understanding of this complex condition by gaining continuous and real-time insights through digital phenotyping into the behavior of autistic adults. Our innovative method combines cross-sectional and longitudinal data gathering techniques to investigate and identify digital phenotypes related to BRs in autistic adults, diverging from traditional approaches. We incorporate ecological momentary assessment and time series data to capture the dynamic nature of real-life events for these individuals. Statistical techniques, including multivariate regression, time series analysis, and machine learning algorithms, offer a detailed comprehension of the complex elements that influence BRs. Ethical considerations and participant involvement in the development of this method are emphasized, while challenges, such as varying technological adoption rates and usability concerns, are acknowledged. This innovative method not only introduces a novel vision for evaluating BRs but also shows promise in integrating traditional and dynamic assessment approaches, fostering a more supportive atmosphere for autistic individuals during assessments compared to conventional methods.

## 1. Context

**Figure 1:**
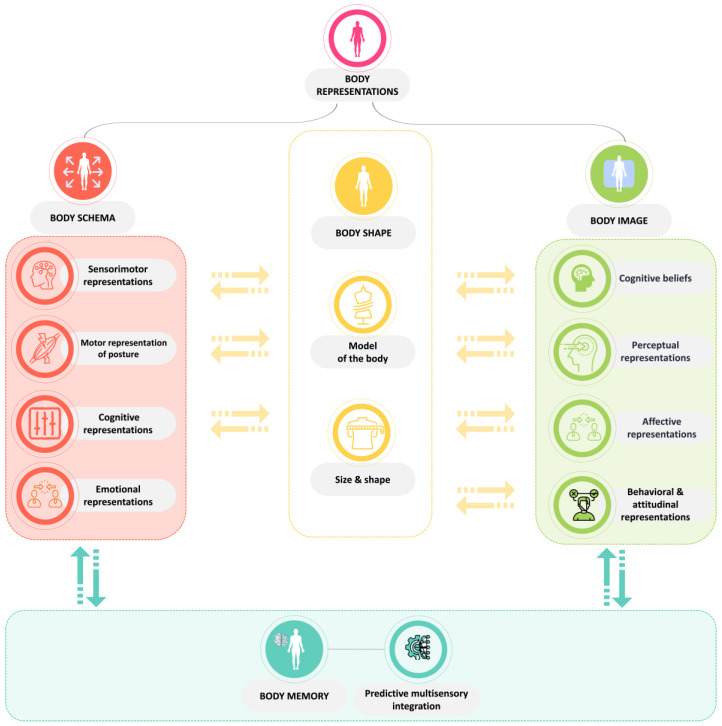
Understanding of the multidimensional aspect of body representations.

Autism spectrum disorder (ASD) stands as one of the most incapacitating developmental disorders, imposing a substantial economic burden on both patients and the healthcare system [1]. While the semantics of ASD remain a contentious topic in research, highlighting the importance of ongoing debate, the terminology employed in this article adheres to that traditionally used in the current literature. Any controversy surrounding it is beyond the scope of our discussion [2]. We define autistic individuals as part of the neurodiversity spectrum, diverging from neurotypical standards not as a disorder but as a representation of the natural variation within humanity [3]. They share certain characteristics, including distinct patterns in social communication and interaction, as well as restricted and repetitive behaviors, interests, or activities [4]. Accurately estimating the prevalence of ASD is crucial. Globally, 1 in 100 children [1] is autistic, and in the United States, approximately 5,437,988 individuals aged 18 and older are autistic [5]. The complex nature of their neurodiversity presents unique challenges for healthcare providers, educators, and caregivers in evaluating their abilities and, subsequently, delivering effective interventions and support services [6]. Within these challenges, clinicians often struggle to accurately assess opportunities and symptomatology and their nuanced yet profound impact on daily life, quality of life, and autonomy. Many autistic adults encounter significant difficulties related to daily life activities, such as personal hygiene, meal preparation, and money management [7,8], alongside more complex issues related to body representations (BRs) [9,10,11]. BR is an umbrella term encompassing both body schema and body image (Figure 1). Body schema refers to a precise sensorimotor representation of postural and structural bodily properties intended to guide the optimal planning and control of actions that are essential for coordinating movements and spatial awareness [12]. Conversely, body image is a multidimensional self-construct representing the visuospatial and conceptual representation of a broad range of bodily properties. Its aim is to describe the body and encompasses feelings of satisfaction or dissatisfaction with one’s physical appearance [12].

By shaping how individuals perceive, experience, and interact with their bodies, BR significantly influences various aspects of daily life, including social interactions, interpersonal relationships, and self-care. This multidimensional construct, which includes cognitive beliefs, perceptual aspects, and affective responses, is crucial for emotional well-being. In fact, the relationship between BRs and mental health is fundamental, extending beyond basic physical self-awareness to encompass one’s subjective self-perception and emotional state. Discrepancies in BRs can give rise to issues like low self-esteem and self-confidence, distorted body image, and difficulties in interpersonal relationships that are often associated with mental health challenges, such as anxiety, depression, and physical health challenges, leading to avoidance behaviors, consequently affecting their academic and/or professional performance [13,14,15]. Challenges in BRs in autistic individuals can manifest as difficulties with motor coordination, spatial awareness, and sensory integration that affect various aspects of daily life. These challenges can significantly impact an individual’s self-esteem, daily life activities, autonomy, quality of life, and mental health [13,14,15,16].

Recognizing and addressing these differences is crucial for cultivating a positive body image, strengthening the body schema, and supporting mental health. Scientific research underscores the importance of interventions aimed at promoting body acceptance and mindfulness to mitigate the mental health risks linked to negative BRs [14,15]. However, to our knowledge, no studies have integrated the multidimensional aspect of BRs in a multisensory environment using multisensors to assess or promote intervention with autistic adults.

Therefore, evaluating BRs is of the utmost importance and covers not only the spatial representation of the body but also the subjective perception of one’s own self [17].

Consequently, there is an urgent need to not only develop an effective multidimensional assessment for identifying autistic adults but also to enhance the understanding of BRs and its influence on autonomy, quality of life, and daily life skills. Such advancements are crucial for facilitating the provision of more tailored care and interventions. Currently, instruments tailored for autistic adults are fewer in number and less rigorously validated compared to those intended for children and adolescents [18] and are mostly limited to the evaluation of a single component of BRs. For instance, screening for autistic toddlers can be performed through the administration of specific standardized, evidence-based tools such as the Modified Checklist for Autism in Toddlers (M-CHAT) [19], the Infant Toddler Checklist (ITC) [20], and the Screening Tool for Autism in Toddlers (STAT) [21]. Additionally, diagnostic assessments like the Autism Diagnostic Observation Schedule (ADOS) [22], the Autism Diagnostic Observation Schedule-2nd Edition (ADOS-2) [23], and the Childhood Autism Rating Scale-2 (CARS-2) [24] are frequently used for children and adolescents. In contrast, access to diagnostic assessments for adults is often hindered by significant limitations. International guidelines suggest integrating clinical observation, (semi-)structured interviews, self-reports, and hetero-anamnesis of developmental history to make a diagnosis [25]. In addition, the majority of current assessment tools are structured as questionnaires that primarily rely on cross-sectional reporting and are designed with singular objectives in mind. Consequently, these tools fall short of providing a holistic evaluation of autistic individuals. This limitation underscores the need for more comprehensive approaches that can capture the multifaceted nature of autism spectrum conditions over time. Additionally, many of these assessments are conducted in a supervised context, failing to consider the real-life circumstances of the individuals being evaluated. Issues such as recall bias and lack of ecological validity highlight the challenges in accurately assessing BRs in autistic individuals since the notion of BRs remains a highly complex and multidimensional concept. Moreover, obtaining information from informants, especially parents, and accessing early developmental history can pose significant obstacles. These challenges complicate understanding the prognostics of the neurodivergence in daily life skills and autonomy [18]. Traditional methods mainly consist of supervised assessments conducted at a single point in time, which could fail to detect symptoms hidden by developmental changes, acquired coping mechanisms, motivation, or the existence of concurrent mental health and neurodevelopmental conditions. The intricate and complex nature of real-life dynamics often involves variability in individual-level determinants that are challenging to assess cross-sectionally [26,27].

Given the aforementioned limitations in the evaluation of BRs in autistic adults, there is an urgent need to develop (semi-)automated and more ecologically valid evaluation tools. Our primary aim is to introduce a novel method to assess BRs by integrating traditional evaluation methods with adaptive, dynamic assessments. This approach is designed to create a more supportive environment for individuals with ASD, advancing beyond the limitations of current standard practices to deliver more precise, individualized interventions [28]. A secondary aim is to determine how such an adaptive and dynamic assessment method can enhance the evaluation of BRs in autistic adults and finally to determine how BR influences autonomy, quality of life, and daily life skills in this population. Our hypothesis is that this more comprehensive assessment will significantly improve the accuracy and ecological validity of BR evaluations in autistic adults, leading to a deeper understanding of BR’s impact on autonomy, quality of life, and daily life skills.

## 2. New Paradigm Opportunity: The Digital Phenotyping Revolution

In response to the current limitations of BR’s evaluation, digital phenotyping (DP) emerges as a promising alternative. DP, which originated in 2015, entails gathering observable and quantifiable attributes, traits, or behaviors of a person, involving the real-time quantification of an individual’s characteristics using data from personal digital devices [29]. It is based on the interactions between a patient and their environment in the digital realm [30]. DP utilizes data collected from personal digital devices continuously, unsupervised, and in real-time to quantify an individual’s behavior within their natural context [31]. This innovative approach offers more ecologically valid and dynamic assessments, potentially revolutionizing our understanding of the complexities underlying BRs in autistic adults and how it influences their daily life activities. Contrary to conventional clinical evaluations, which heavily rely on subjective observations and self-report measures—as presented above—DP harnesses the power of digital technologies to gather objective real-time data on individuals’ behavior, cognition, and physiological responses in their natural environments [32,33,34].

In recent years, there has indeed been a notable paradigm shift in the field of medical evaluation, moving away from the conventional clinic-based model toward a more patient-centric approach conducted within the comfort of patients’ homes thereby bridging multi-center and multidisciplinary methodologies [35,36]. This transition marks a significant opportunity to revolutionize the way we assess and monitor patients. In the realm of ASD assessment, the emergence of DP heralds a groundbreaking shift in diagnostics, particularly for adults.

In fact, accessing accurate and comprehensive assessments in ASD has long been hindered by numerous barriers, including difficulties in verbal communication, sensory sensitivities, and the inherent challenges of self-reporting [37]. Moreover, the conventional clinic-based approach may exacerbate these obstacles, leading to incomplete or inaccurate evaluations that fail to capture the nuances of their neurodivergent and neurodevelopmental profile [38,39].

By leveraging advancements in technology and telemedicine, and by transitioning to a home-based DP framework, clinicians and researchers can overcome many of these limitations, offering a more holistic and ecologically valid assessment of autistic adults’ functioning [40]. Evaluating BRs within the context of DP can provide valuable insights into the holistic functioning of autistic adults, ultimately informing targeted interventions and support strategies to enhance their overall quality of life. Through continuous monitoring and analysis of these factors using wearable devices, smartphone applications, and other digital tools, clinicians can identify patterns, triggers, and interventions to address challenges related to BRs in real time. They can then offer more personalized and effective assessments tailored to the specific needs and preferences of autistic adults, ultimately fostering greater autonomy, well-being, and participation in daily life activities [41].

With our proposed new method, we aim to deepen our comprehension and assistance for autistic individuals by investigating novel technological approaches to evaluate BRs.

## 3. Data Collection Pipeline

**Figure 2:**
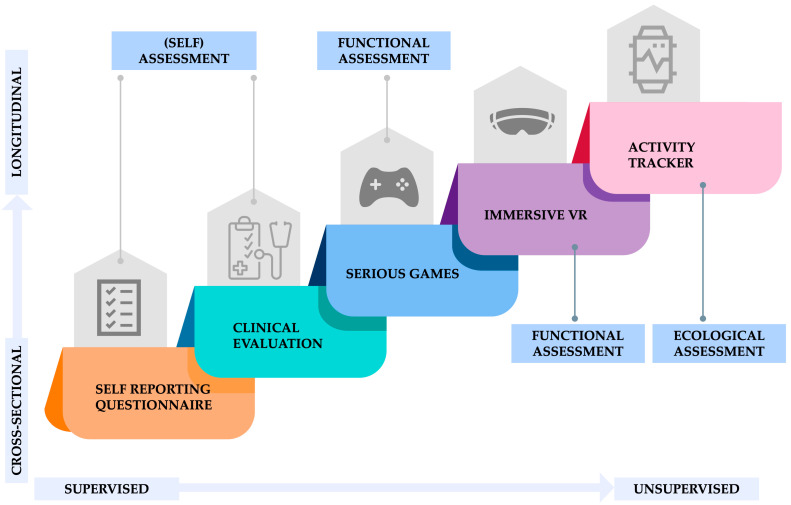
Multidimensional and multiple sensors assessment of BRs in autistic adults.

A data collection pipeline was specifically developed to evaluate BRs in ASD, employing a combination of supervised and unsupervised assessments in both cross-sectional and longitudinal settings using a stepwise approach. Figure 2 illustrates the different steps of the data collection process. Data collection employs a sophisticated array of tools, including wearable sensors and mobile tracking applications, which continuously capture a wide range of behavioral, physiological, and environmental data. This setup enables a detailed analysis of the temporal and spatial dynamics of BRs in autistic individuals. Each step builds upon the previous steps, gradually increasing the multidimensional evaluation by adding complexity and more quantitative, robust assessment.

A brief description of the different steps is presented in Table 1, while a complete description of the tests and the rationale behind them is provided in the following section.

### 3.1. Self-Reporting Questionnaire

The first step of the data collection process involves the administration of a self-report questionnaire aiming to evaluate self-representation and perception of BRs. This questionnaire includes questions to evaluate participants’ awareness of body schema and body image. Various factors, such as general information (age, gender, height, marital status, level of education, living arrangement, income level, self-rated autonomy level, sleep patterns, etc.), as well as aspects of the psychomotor domain, including well-being (empathy, emotional regulation, depression, stress, anxiety, emotional loneliness, etc.), cognitive functioning (communication, adaptive strategies, etc.), motor functioning (physical activity, etc.), and BRs (different types of representations of body schema and body image shown in Figure 1) will be evaluated. To accommodate attention span variability and neurodivergence-related difficulties, the questionnaire will utilize a randomized sequence of questions and include a mix of closed-ended and multiple-choice questions. Additionally, a neuropsychological assessment will be conducted to provide supplementary data [42].

### 3.2. Clinical Evaluation

In addition to the self-report questionnaire, behavioral observational data are obtained through structured tasks and activities designed to evaluate BRs, movement coordination, and sensory integration. The data collection process involves the administration of the BR assessment, which draws upon current evidence to compile assessments concerning BRs in ASD.

The assessment of BRs will be conducted comprehensively through clinical evaluations administered by experienced and trained psychomotor therapists, psychologists, and physiotherapists. This assessment encompasses motor skills, cognitive functioning, body image, and body schema tasks, providing a holistic understanding of an individual’s overall body awareness.

Furthermore, diverse data will be gathered during the BR assessment using gold-standard questionnaires, such as the Body Image Questionnaire-20 (FKB-20) [43], the Movement Imagery Questionnaire-3 (MIQ-3) [44], and the QUIMOT [45]. The MIQ-3 comprises 12 items designed to assess an individual’s ability to imagine four different movements using internal visual imagery, external visual imagery, and kinesthetic imagery [44]. Another assessment tool, the Body Image Disturbance Questionnaire (BIDQ) [46], consisting of seven questions, specifically focuses on body image disturbances. In addition to these questionnaires, general scales related to mental health, such as emotion regulation and empathy scales, will be utilized. Additionally, the Motor Imagery Questionnaire—QUIMOT (QUIMOT) is an adaptation of the Praxis Imagery Questionnaire [47] and has the following four initially constructed subscales: Kinesthetic Scale (questions regarding the preferential use of a body part), Position Scale (questions concerning the position of body parts), Action Scale (relative to one type of movement compared to another), and Object Scale (relative to the characteristics of the object used during the action). These questionnaires will be used as tools to validate the Body Representation Assessment as a clinical evaluation to assess BRs in ASD.

### 3.3. Serious Games

While serious games (SG), which are games designed with a primary purpose other than pure entertainment [54], are currently mostly used for rehabilitation purposes, there is a growing body of evidence suggesting that SG, combined with sensors and markerless technology, can be used to evaluate patients while they are performing their rehabilitation exercises. Such evaluation can be performed for both motor [55] and cognitive functions [56]. Current evidence indicates that assessing cognitive abilities using mobile games demonstrates sensitivity to age-related changes in scores, whereas other evidence leans on the effectiveness of detecting motor impairment, thereby highlighting the potential to integrate SG to perform functional evaluation [55]. We will use an SG platform specifically developed for rehabilitation purposes: STASISM. This platform is the result of a collaborative co-creation effort, bringing together expertise from a variety of fields, such as therapy, medicine, software engineering, and data analysis. The development was guided by feedback from real-world users, ensuring its relevance and efficacy [48]. While alternative assessments might struggle to maintain the engagement of autistic individuals, particularly with concentration and sensory sensitivities, SG offers unique advantages in terms of engagement, ecological validity, customization, and data collection. By leveraging the interactive and immersive nature of SG, proprioception assessment in autistic individuals can be made more effective, meaningful, and enjoyable, potentially leading to more accurate and reliable assessment outcomes.

Various sensors, such as balance boards, accelerometers, or markerless cameras, can be used, and the data can be recorded and later analyzed to facilitate customized rehabilitation activities and remote patient monitoring, offering real-time feedback to enhance the effectiveness of assessments. This is crucial for autistic adults who may face challenges with traditional methods or struggle with in-person assessments due to sensory sensitivities or communication difficulties.

The platform also incorporates validated assessment tools for both upper limb, trunk, and balance analysis, ensuring accuracy in tracking patients’ advancement and progress, thereby facilitating data-driven decision-making by healthcare professionals and enhancing the accuracy of BR evaluation. Moreover, the approach facilitates evidence-based decision-making for future rehabilitation plans through the incorporation of rehabilomics, which is a novel framework that combines the systematic data collection of rehabilitation-related traits with interdisciplinary biomarker analysis. This integration deepens our understanding of disability biology, function, prognosis, and treatment [57].

### 3.4. Immersive Virtual Reality

Immersive virtual reality (VR) systems encompass technologies that completely engage users’ senses within a simulated environment [58]. This immersion is typically achieved through the use of a head-mounted display, although in some cases, large curved displays with panoramic views are employed [59]. Within VR setups, users primarily engage with the virtual environment via various input devices, such as controllers, joysticks, or motion capture cameras. Due to advancements in technology, VR is increasingly being utilized in clinical settings. While VR has been extensively utilized in different types of treatments, including clinical psychology [60], neuropsychology [61], and cognitive and motor rehabilitation [62,63], its role in the assessment of autistic individuals, particularly regarding BR, remains less well-explored [49]. Nevertheless, given the recent enhancements in VR technology—specifically in terms of fidelity, immersion, and accessibility—the prospect of evaluating BRs in autistic individuals appears promising. VR’s compatibility with the unique characteristics of ASD, including its ability to offer customizable environments, control over stimuli, and engagement in safe yet challenging tasks, highlights its potential effectiveness. Furthermore, VR provides ecological validity to simulated situations, allowing for a more accurate observation of users’ reactions. Many studies indicate promising feasibility and acceptability of VR interventions among autistic individuals, suggesting its utility in assessments to gain insights into an individual’s psychomotor profile and holistic understanding of their abilities and needs [64].

In addition to providing an immersive experience that allows for modifications of the body (such as altering the size and length of body segments and body composition), environmental factors, and social components, another notable and advantageous aspect of VR is its ability to monitor upper limb mobility and functions without requiring patients to be equipped with external sensors [65]. It is, however, worth noting that most of the systems are currently focusing on stroke [66], while such a type of evaluation has not been performed yet in ASD.

Even more interestingly, when it comes to autistic individuals, VR systems can be used to collect physiological data using pupillometry [67,68]. Briefly, the pupillary light reflex (PLR) presents a promising avenue for the bedside assessment of alterations in autonomic nervous system activity [69]. Simplistically, the autonomic mechanism governing pupil dynamics can be delineated as follows: pupil constriction is facilitated by parasympathetic activation of the circular sphincter pupillae muscle, while pupil dilation is mediated by sympathetic activation of the radial dilator pupillae muscle [70]. For instance, an increase in pupillary constriction velocity serves as an indicator of heightened parasympathetic tone [71]. The parasympathetic system predominantly influences pupil constriction in response to light stimuli. Following stimulus cessation, both parasympathetic and sympathetic nervous systems contribute to the initial phase of dilation, with the sympathetic nervous system primarily influencing the latter phase of dilation. Pupillometry will be combined with heart rate analysis, particularly, heart rate variability (HRV) taken into account while moving [72], to assess autonomic measures when participants will be immersed in various virtual situations and conditions.

To gain deeper insights into the functioning and interaction of autistic individuals within virtual environments, we aim to analyze body movements and bodily responses in a multimodal VR experience, integrating diverse sensory stimuli. Our focus lies on examining the sense of embodiment, which encompasses aspects such as self-location, a sense of agency, and a sense of ownership. Tactile experiences will be utilized to assess this sensation and its impact on movement behavior. By investigating how autistic adults utilize proprioception, their sense of self, and their perception of others within virtual settings, we aim to evaluate the significance of bodily feedback for fostering self-awareness and agency in daily life activities.

Given that motor impairment is recognized as a core feature in ASD, impacting adaptive behavior and symptom severity, the utilization of low-cost motion capture and VR game technologies holds promise for providing a better understanding of BRs in autistic adults.

### 3.5. Activity Trackers

The last step of the evaluation consists of an unsupervised assessment of patients in their daily environment. The utilization of smartwatches will facilitate the comprehension of BRs among autistic adults within an ecological assessment framework. Over the course of a two-week trial, participants will engage in continuous monitoring by wearing a non-intrusive sensing device 24/7 [31]. This device will record various parameters, including stress levels, physical activity, step count, calorie expenditure, heart rate, number of floors climbed, moderate to vigorous activity, and sleep patterns. In addition, an ecological momentary assessment (EMA) will be used to better understand the context in which the data related to activity level are collected (~metadata). The EMA’s data will be collected in a structured manner and repeated using intensive sampling to study individuals’ behavior, cognition, affect, context, and other experiences in real-time and ecological settings [53]. This comprehensive approach aims to offer insights into the impact of BR on daily living activities, as well as its influence on individuals’ emotional states and habits. By gathering and analyzing these data, our objective is to gain a deeper understanding of how BR manifests in the lives of autistic adults and its potential effects on their needs and behaviors.

## 4. Data Management and Analysis Framework Development

**Figure 3:**
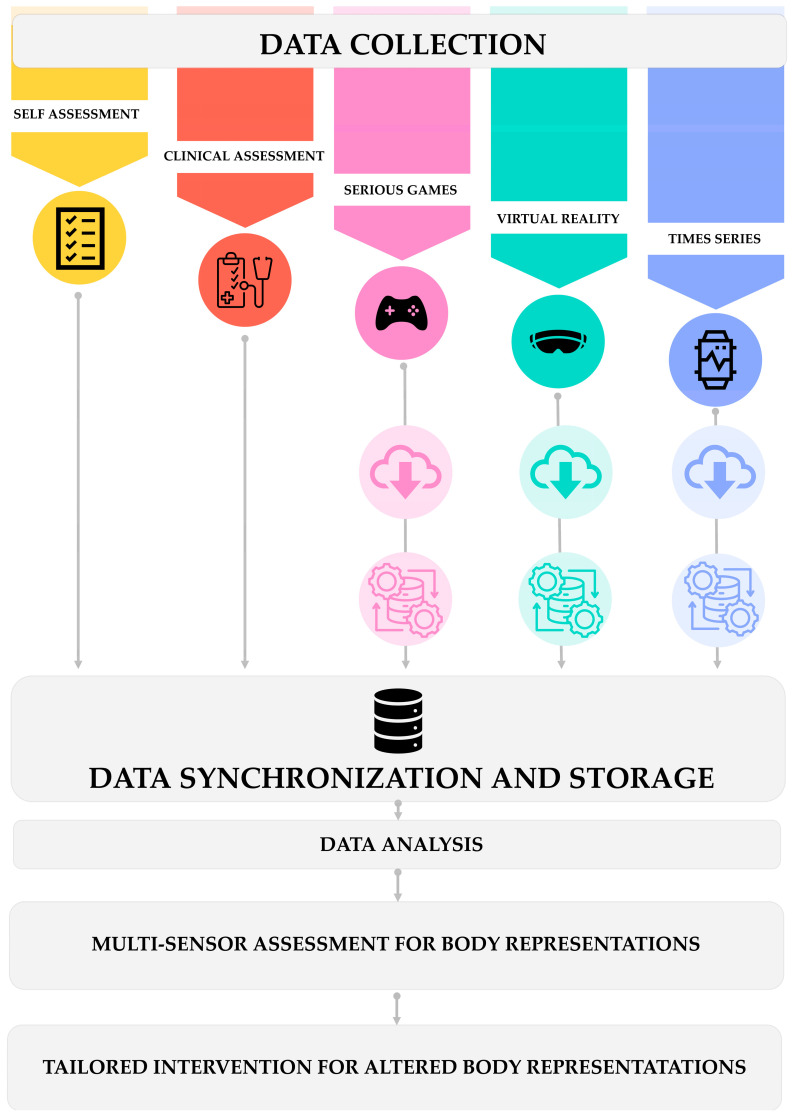
Data management and analysis framework.

Based on the outlined data collection methods, we present our proposed methodology for setting up a proper framework to store, synchronize, and later analyze the data (Figure 3). We have implemented a state-of-the-art data integration platform that utilizes machine learning algorithms for data cleaning and transformation, ensuring compatibility across different device platforms and data formats. The platform features advanced algorithms for anomaly detection and correction, significantly reducing integration errors and improving the reliability of multisource data aggregation. This step is crucial for linking the technological aspects, such as the various sensor evaluations, with clinical applications and research. Developing such an integrated platform is essential for fully exploiting the potential of the collected data. Without such a platform, the analysis would be restricted to individual evaluations, limiting the opportunity to leverage the different evaluations performed to gain deeper insights into ASD.

### 4.1. Data Collection Standardization

In order to ensure high-quality data, it is of the utmost importance to develop uniform templates for gathering data from every source to guarantee uniformity and interoperability among all data categories. This entails establishing data structures, schemas, and metadata needs for self-reporting surveys, clinical assessments, SG, VR, and activity trackers.

### 4.2. Integration Architecture Design

Robust data mapping and transformation techniques are crucial in developing this framework. This entails establishing detailed data mapping rules to aid in converting raw data gathered from different sources into a standardized format appropriate for integration [73]. The rules specify how each data element from self-reporting questionnaires, clinical examinations, virtual reality simulations, and activity trackers maps to particular fields in the integrated dataset.

Several data transformation methods, such as data cleaning, normalization, aggregation, and enrichment, will be integrated into the platform. Data integrity, accuracy, and completeness are guaranteed throughout the integrated dataset using these methods.

Data quality assurance procedures will be applied with data transformation to identify and rectify mistakes, inconsistencies, and anomalies in the combined dataset [74].

Metadata management procedures will also be implemented to document and monitor metadata linked to connected datasets. Metadata repositories store data on data sources, mappings, transformations, and lineage to support data governance, lineage tracking, and impact analysis [75].

### 4.3. Data Synchronization and Storage

Data synchronization and storage are crucial elements, forming the basis for capturing, storing, and syncing varied data from different sources. The architecture guarantees data consistency, availability, and durability by using scalable and robust storage technologies and synchronization techniques [76]. This will allow researchers and clinicians to extract actionable insights and make clinical decisions using integrated datasets. Integrated data will be stored on a cloud-based storage service (Google Cloud Storage).

### 4.4. Data Analysis

Various statistical methods will be tested to identify optimal combinations that yield maximum clinical insights from our dataset.

#### 4.4.1. Exploratory Data Analysis

Exploratory data analysis is performed to obtain an initial comprehension of combined datasets, recognize trends, patterns, and outliers and develop hypotheses for more research.

Descriptive statistics and data visualization techniques like scatter plots, histograms, box plots, and heatmaps will be used to visually represent data patterns, trends, and anomalies, aiding in the intuitive comprehension and interpretation of combined datasets [77].

Interactive dashboards will be created in Power BI to generate interactive data representations and investigate complex relationships among these datasets.

#### 4.4.2. Statistical Modeling and Analysis

Statistical modeling approaches will be used to discover correlations and associations in the combined datasets, allowing for hypothesis testing, prediction, and inference. This entails using regressions, time series analysis, mediation analysis, and multivariate analysis tools to measure correlations and deduce causality from the combined data [78].

Time series analysis approaches, such as autoregressive integrated moving average (ARIMA) models or exponential smoothing methods, will be used to assess patterns and trends in time series data gathered from the activity tracker (e.g., number of steps, heart rate, sleep indicators) [79]. A principal component analysis (PCA) and factor analysis will be used to discover hidden variables and simplify complex datasets, making it easier to recognize patterns and comprehend the data [80].

#### 4.4.3. Artificial Intelligence: Between Machine Learning and Predictive Analytics

Next, we apply machine learning algorithms, which are a subset of artificial intelligence, to create predictive models, categorize data, group related instances, and derive insights from combined information [81]. To do so, different models will be used and tested according to the quantity and the complexity of the collected data, such as supervised learning, unsupervised learning, and reinforcement learning methods, to uncover concealed patterns and correlations among combined data [82].

Supervised learning methods like decision trees, random forests, support vector machines, and neural networks are utilized to predict outcomes or categorize occurrences in datasets by being trained on labeled data [83]. Unsupervised learning algorithms, such as k-means clustering and hierarchical clustering, are used to detect inherent groups or clusters in the combined data [84].

Finally, we will test reinforcement learning methods using Markov decision processes [85]. This method is used to enhance decision-making and control actions by utilizing feedback from various data sources. These methods facilitate self-directed learning and adjustment to evolving settings or situations, thereby improving the flexibility and reactivity of data-based systems.

### 4.5. Feedback Mechanisms

It is crucial to include feedback mechanisms to continuously improve the system. This iterative strategy includes gathering feedback from different stakeholders, such as physicians, researchers, and autistic individuals, to consistently improve data collection methods, integration processes, and analytical capabilities.

Clinicians are essential in evaluating the efficacy and user-friendliness of the framework in clinical environments. Their input on the usability, relevance, and clinical usefulness of the combined data and analytical results is crucial for enhancing the framework’s design and functionality. Conducting regular surveys, interviews, or focus group discussions can help gather information from clinicians about their experiences, issues, and suggestions for improvement [86].

Researchers enhance the framework by assessing its scientific validity, reliability, and robustness. Researchers’ feedback might pinpoint areas needing methodological improvement, algorithm optimization, and data quality enhancement. Engaging with scholars through workshops, collaborative projects, or scientific conferences enhances information sharing and fosters ongoing enhancement of the framework [87].

Last but not least, feedback from autistic individuals is essential in meeting their specific requirements, preferences, and experiences [88]. Involving autistic individuals in the co-design process via participatory research methods like co-creation workshops, user testing sessions, or advisory panels helps create user-centered solutions that cater to their unique needs and objectives [89]. Input from individuals with autism enhances the accessibility, usability, and inclusivity of the framework, eventually improving its relevance and impact.

### 4.6. Scalability and Flexibility

In the context of the rapid development of the technology, the scalability and flexibility of the platform are important to maintain the system and keep it up to date [90]. The framework has been created with modular components and architectures that can easily be adjusted to evolving clinical requirements and support future expansions.

Currently, our proposed pipeline is composed of five different and interchangeable modules. Each component has a distinct purpose, presented in Table 1, including data acquisition, processing, storage, or analysis, and can be expanded or substituted without impacting the entire system. The modular design enhances flexibility, extensibility, and reusability, enabling the easy addition of new functionalities or the modification of current ones with minimal disturbance.

By following this methodology, this framework can effectively collect, integrate, and analyze data from diverse sources to gain insights into BR in autistic adults and its impact on their needs and behaviors.

## 5. Challenges and Potential Pitfalls

While the proposed solution offers considerable potential, numerous challenges remain that must be addressed to fully leverage this methodology. These challenges can be categorized across the following three distinct levels: the participants, the technology, and the data.

### 5.1. Patients’ Perspectives

Autistic individuals share common traits related to social communication and interaction, as well as a complex sensory profile characterized by hypersensitivity, hyposensitivity, or sensory-seeking behaviors. This intricate sensory profile poses significant challenges in a multisensor approach, as the sense of touch can disturb them during the assessment [91], and the immersive experience with VR can also be overwhelming for these patients [92]. Additionally, wearing a smartwatch in the last phase of the assessment can further exacerbate sensory issues.

Furthermore, the symptomatology related to social communication and interaction can also impact the multidimensional approach, as repeated assessments at various time points may be exhausting for them. Receiving many messages throughout the day can be cognitively challenging in the context of EMA, and the use of technology might also be challenging for participants, especially when it comes to wearing a VR headset, as it may cause discomfort or anxiety. Practitioners should be aware of these challenges and be equipped with strategies to support individuals throughout the assessment process, taking into consideration potential comorbidities. In this study, comorbidities will be controlled during the first phase of the assessment (questionnaire) while considering DSM-5 TR criteria. The use of activity trackers and EMA poses additional challenges when assessing autistic individuals. While activity trackers offer valuable insights into physical activity levels, sleep patterns, and other behaviors, autistic individuals may encounter discomfort or feel intruded upon when wearing such devices. Additionally, their ability to continuously wear a watch for a prolonged period, which is typically required for accurate data collection spanning over 2 weeks, may be compromised. Furthermore, autistic individuals might become overly fixated on the technology aspect of the watch, leading to distractions from other tasks or activities.

### 5.2. Technological Challenges

EMA involves collecting real-time data on mood, behaviors, and environmental factors through frequent prompts on mobile devices. However, this method can prove overwhelming for autistic adults, particularly those who are sensitive to changes in routine or stimuli. Moreover, the abstract nature of some EMA questions may present challenges in comprehension and accurate responses for autistic adults [93].

Therefore, it is imperative to carefully consider and adapt these assessment methods to accommodate the unique needs of autistic individuals. This includes strategies to mitigate discomfort, minimize distractions, and ensure the comprehension of EMA questions. By doing so, meaningful and accurate data collection can be achieved without causing undue distress or discomfort. Ensuring device compatibility is crucial to prevent exclusion based on device preferences. Taking into consideration diverse sensory sensitivities and preferences is essential for engagement and usability.

### 5.3. Data-Related Challenges

One significant challenge is balancing the advantages of using big data with the drawbacks of potential data quality issues. While big data offer vast amounts of information, they frequently lack the precision and cleanliness essential for correct analysis [94]. On the other hand, while clean data ensure both accuracy and reliability, they might be limited in scope, hindering this study’s findings. In addition, integrating data from different sources requires careful methodology, which can be challenging due to differences in data formats and structures. The lack of synchronization can present inconsistencies in the way findings were analyzed. Furthermore, we should take into consideration the risk of overfitting or underfitting models when integrating data, which could potentially lead to misrepresentative conclusions [95].

## 6. Discussion

Autistic individuals are neurodivergent and present a heterogenous profile exhibiting variability that spans across all ages, genders, and IQ levels [26,27]. Although the diagnosis rate of autistic adults has exponentially increased, ASD is still under-recognized and poorly diagnosed. Patients not only lack adequate care but also face a shortage of mental health services. Current services often fail to address and understand their comprehensive needs and specific requirements [27]. The significance of a multisensor evaluation in autistic adults cannot be overstated, particularly when combining both cross-sectional and longitudinal assessments. Our innovative method bridges a critical gap in the field by providing a more comprehensive understanding of BR and its implications for daily life activities. By assessing individuals over time, we gain insights into the dynamic nature of their experiences and how these evolve over the lifespan. This holistic perspective enables us to better tailor interventions and support strategies to meet the diverse needs of autistic individuals, thereby fostering improved outcomes and quality of life [96]. In fact, this is a paradigm-shifting strategy in the field of rehabilitation and presents many benefits over conventional in-person care. This proposed robust method can help autistic individuals understand their BRs and their effects on daily life activities. The novelty of this assessment lies in its multisensor approach, which addresses challenges faced in traditional assessments and offers a potentially effective solution for patient care. This holistic assessment aligns with the latest theoretical frameworks in rehabilitation science based on patient-centered approaches promoting patient involvement in the awareness of their BRs [97].

A key element of this multidimensional assessment is related to the ongoing tracking of autistic individuals’ development for a period of two weeks, providing insights not only into their daily activities but also their autonomy, well-being, and body awareness. This information facilitates the development of personalized rehabilitation protocols by integrating physiological, kinematic, and environmental data.

Healthcare professionals can adopt this new assessment shift as a useful tool to holistically understand their patients.

However, several limitations need to be addressed. Despite its potential benefits, the adoption of this new assessment approach among healthcare professionals remains limited, particularly due to reservations about the multisensor approach. Emerging technologies, such as SG, VR, and activity tracking, raise ethical concerns regarding patient privacy [98]. The potential for hacking and compromising patient history underscores the need for robust ethical considerations, especially in home settings where evaluations are conducted. Intrusion into personal privacy raises valid concerns regarding consent, confidentiality, and autonomy, emphasizing the importance of ethical guidelines in research and clinical practice. It is important to use the findability, accessibility, interoperability, and reuse (FAIR) of digital datasets principle when collecting and analyzing data [99]. This framework requires the creation of unique and deidentified metadata for easy discovery, open or federated access points for accessibility, comprehensive data sharing for interoperability, and data with accurate attributes under clear usage agreements for reusability. In addition, concerns about the ethical implications of depriving individuals of evidence-based treatments for research further compound these challenges. Striking a balance between data collection and respecting individuals’ rights and boundaries is imperative, necessitating vigorous ethical guidelines and protocols.

Moreover, financial constraints and resistance to change among clinicians and patients may pose additional hurdles. The technical complexity of implementing such evaluations requires ongoing training and collaboration between researchers, clinicians, and engineers. Cultural differences in user preferences and perceptions of healthcare also contribute to challenges in acceptance and implementation [100].

While multidimensional evaluations offer valuable insights into the BRs of autistic adults, addressing the associated challenges is essential for their effective implementation.

Given the diverse ways in which autism manifests, our method provides an in-depth analysis of how multidimensional constructs affecting daily life activities, quality of life, and autonomy interact in participants with inherently heterogeneous profiles. This approach specifically addresses the unique characteristics of each individual, allowing us to better understand how these factors combine to shape their experiences. Studies have shown atypical BRs in autism [101], and challenges faced in this regard often go unaddressed, contributing to higher mortality rates among autistic individuals, particularly due to suicide [102]. Understanding how these BR challenges impact daily activities, self-esteem, autonomy, quality of life, and mental health in autistic individuals is therefore essential. It is important to emphasize shifting our assessment methods to include DP, which can provide a better understanding of the behavioral aspects of semiology and its symptomatology. This pioneering method proposes a combination of both quantitative and qualitative approaches, ensuring a robust and holistic understanding of the issues at hand.

## 7. Conclusions

The challenges surrounding the understanding of BRs in ASD underscore the critical need for more effective assessment protocols. Traditional methods often fall short in capturing the complexities of autistic individuals’ experiences, particularly regarding BR and its impact on daily life activities.

The emergence of DP presents a promising alternative, offering dynamic and ecological assessments that better reflect real-world dynamics. By leveraging personal digital devices to collect continuous, unsupervised, and real-time data, DP holds the potential to revolutionize diagnostic procedures for autistic individuals. Moreover, the transition from clinic-centric to home-based evaluation further enhances the potential of DP in assessing BRs. This paradigm shift allows for a more patient-centric approach, conducted within the familiar environment of the individual’s home.

By integrating assessments of BRs into DP frameworks, clinicians and researchers can gain valuable insights into how autistic adults perceive and interact with their bodies in daily life. These insights can inform personalized interventions and support strategies, ultimately enhancing the overall quality of life for autistic adults. Through the continuous monitoring and analysis of these factors using wearable devices, smartphone applications, and other digital tools, clinicians can identify patterns, triggers, and interventions to address challenges related to BRs in real time.

This holistic approach enables clinicians to offer more personalized and effective interventions tailored to the specific needs and preferences of autistic adults, fostering greater autonomy, well-being, and participation in daily life activities.

## Figures and Tables

**Table 1:** Concise overview of the proposed methods for evaluating BRs in autistic adults.

Variables	Description	Type	Target	Added Value
Self-Reporting Questionnaire	Administered to evaluate self-representation and perception of BRs, it includes different types of representation of body schema and image and understanding the complex interplay of BRs in autistic adults.	Self-reported short sentences questionnaire	General information Psychomotor domain Perception of sensorimotor representations Perception of motor representation of posture Perception of cognitive representations Perception of emotional representations Perception of cognitive beliefs Perception of perceptual representations Perception of affective representations Perception of behavioral/attitude representations	Accommodates attention span variability and neurodivergence-related difficulties; Utilizes randomized question sequence; Provides additional data through neuropsychological assessment [42]
Clinical Evaluation	Involves structured tasks and activities and a clinical BR assessment by experienced therapists and psychologists in order to have a comprehensive assessment of motor skills, cognitive functioning, body image, and body schema	Body Representation Assessment (clinical assessment)	Sensorimotor representation tasks Motor representation of posture tasks Cognitive representation tasks Emotional representation tasks Cognitive beliefs assessment Perceptual representation assessment Affective representation assessment Behavioral/attitude representation assessment	Utilizes gold-standard questionnaires; Provides a holistic understanding of individual’s body awareness [43,44,45,46,47]
Serious Games	Participants will be assessed in a clinical, unsupervised manner to understand their BR performance	STASISM	Upper limb, trunk, balance function BR task and proprioception Visual–spatial ability Navigation skills Attention Motor coordination: gross motor skills and fine motor skills Stress relief	Offers engagement, customization, and real-time feedback; Utilizes advanced analytics for precise motion analysis; Incorporates wearable sensors for unsupervised daily mobility assessments [48]
Immersive VR	Gain insights into the psychomotor profile and holistic understanding of abilities and needs by applying VR systems to assess BRs in autistic individuals. Monitors upper limb mobility and physiological data.	PICO Neo3	Analyze body movement and bodily response Sense of embodiment	Offers customization of environments; Provides ecological validity to simulated situations; Collects physiological data for deeper understanding; Analyzes autonomic measures through pupillometry and heart rate analysis [49,50,51,52]
Activity Tracker	Uses smartwatches for unsupervised assessment of daily activities. Incorporates ecological momentary assessment (EMA) in order to understand the impact of BRs on daily living activities and gain insights into emotional states and habits	Garmin Vivosmart 5	Stress levels Physical activity Step count Calorie expenditure Heart rate Number of floors climbed Moderate to vigorous activity Sleep patterns. Behavior Cognition Affect	Provides continuous monitoring of various parameters; Offers real-time insights into behavior and cognition; Aims to understand BR effects on needs and behaviors [53]

## Data Availability

Not applicable.
